# Structure and validity of the Subjective Traumatic Outlook questionnaire under conditions of continuous trauma

**DOI:** 10.1192/bjo.2026.10981

**Published:** 2026-02-23

**Authors:** Shir Mor-Ben-Ishai, Menachem Ben-Ezra, Yafit Levin

**Affiliations:** School of Social Work, Ariel University, Ariel, Israel

**Keywords:** Continuous trauma, trauma-related subjective distress, subjective trauma outlook, PTSD, CPTSD

## Abstract

**Background:**

This longitudinal study examines the psychometric validity of the Subjective Traumatic Outlook (STO) questionnaire by evaluating its structural consistency and diagnostic performance in a conflict-affected context. The STO was used to measure trauma-related subjective distress at two time points following the terrorist attacks in Israel on 7 October 2023.

**Aims:**

The primary aim of this study was to evaluate the STO as a concise and reliable assessment tool for populations affected by armed conflict.

**Method:**

A nationally representative sample of 4097 participants responded at T1, of whom 2005 completed the study at T2. Data were collected during the ongoing war in Israel. Participants completed the STO alongside validated measures of post-traumatic stress disorder (PTSD (PTSD Checklist for DSM-5) and International Trauma Questionnaire), depression, anxiety and adjustment disorder. Exploratory factor analyses were used to estimate one- to three-factor solutions using robust maximum likelihood estimation. Convergent validity was assessed through bivariate correlations with trauma- related measures. Receiver operating characteristic analyses were conducted to evaluate diagnostic utility for PTSD and complex PTSD per the ICD-11.

**Results:**

Exploratory factor analysis supported a stable two-factor structure across both waves. The STO demonstrated strong internal consistency and stable convergent validity over time. Receiver operating characteristic analyses indicated that the four-item version matched or slightly outperformed the five-item version, suggesting improved parsimony without loss of diagnostic accuracy.

**Conclusions:**

The stable factor structure of the STO and its strong psychometric properties across both waves within a wartime context support its utility for large-scale screening and early detection of trauma-related distress.

The Subjective Traumatic Outlook (STO) is a conceptual framework and screening tool^1^ developed to capture how individuals perceive, interpret and respond to traumatic events. Subjective experiences following trauma exposure may serve as valuable indicators for identification of individuals at risk of developing mental health disorders.^1^ These internal, personal changes often provide a more sensitive measure of the psychological impact of an event than objective criteria alone. The STO has demonstrated the ability to differentiate between post-traumatic stress disorder (PTSD) and complex PTSD (CPTSD) in diverse populations.^2,3^

The theoretical foundation of the STO relates to how individuals perceive pre-trauma self-concepts compared with their current psychological state.^1^ This assessment framework relies on their ability to recognise that a fundamental shift has occurred within themselves following trauma exposure. Drawing from psychodynamic theory, Freud^4^ conceptualised the distinction between ego-syntonic experiences (consistent with self-identity) and ego-dystonic experiences (creating internal conflict and distress). When trauma becomes ego-dystonic, it fundamentally challenges one’s sense of self, generating cognitive dissonance that may manifest as PTSD or CPTSD symptomatology.^5^ This theoretical framework applies particularly to CPTSD, in which persistent affect dysregulation often reflects the ongoing tension between traumatic experiences and core identity structures, with symptom severity fluctuating on the basis of contextual stressors.^6^ The STO operates as a mediating framework that translates subjective evaluations of trauma into indicators of psychological risk by assessing the correspondence between traumatic experiences and the individual’s foundational self-concept.

Janoff-Bulman^7^ developed the World Assumptions Scale (WAS), proposing that traumatic experiences fundamentally disrupt individuals’ core beliefs about the benevolence of the world, meaningfulness and personal invulnerability. Her framework emphasises how the perception of trauma during and after its occurrence shapes post-traumatic pathology. Although both the WAS and STO address the impact of trauma on cognitive schemas, they differ fundamentally in their approach and scope. The WAS focuses on measuring relatively stable, pre-existing world assumptions and their disruption, treating these beliefs as enduring cognitive structures that become violated by trauma, in contrast to the STO. This distinction is crucial; whereas the WAS assesses the content of disrupted beliefs, the STO evaluates the subjective experience of transformation itself, providing insight into the individual’s awareness and interpretation of their post-traumatic psychological shifts.^1^

The ongoing Israeli Palestinian conflict provides a critical context for examination of trauma. On 7 October 2023, Hamas launched a large-scale terrorist attack on Israel, killing more than 1300 people and abducting 229, including infants and the elderly. As the deadliest terror event in the history of Israel,^8^ it led to a sharp rise in psychological distress, with PTSD prevalence nearly doubling (16.2 to 29.8%) and marked increases in anxiety and depression.^9^ These figures underscore the profound mental health impact of the attack, which, as prior research has shown, extends beyond direct victims to affect the broader population through collective trauma.^10,11^

## Aim of the study

The present study aimed to assess the psychometric properties of the STO during continuous trauma, focusing on its applicability in the immediate aftermath of the 7 October attack and amid an ongoing war. Although previous research has demonstrated the predictive capabilities of the STO in prolonged conflicts – for instance, our previous research in Ukraine, which examined the STO with respect to differentiation between ICD-11 PTSD and CPTSD in the context of the Russian invasion – its psychometric validity has not been systematically tested. In this study, we sought to fill that gap by testing the STO as a reliable screening tool for the ICD-11 disorders associated with stress, namely PTSD and CPTSD, during continuous trauma, specifically assessing its factor structures and construct validity as a screening tool for early psychological assessment in populations affected by war-related trauma. Establishing its psychometric robustness at this critical juncture could facilitate its use as a concise and effective measure in conflict-affected populations. Moreover, as the STO has been shown to serve as an effective screening tool for PTSD and CPTSD,^3^ it is important to examine whether a shorter version could retain comparable psychometric properties.

The study focuses on the Israeli general population after the 7 October attacks. We proposed that the STO would demonstrate strong internal consistency and construct validity in measuring subjective trauma perception during continuous trauma. In addition, we expected the STO to show medium-to-large correlations with trauma-related measures, including the International Trauma Questionnaire (ITQ) and PTSD Checklist for DSM-5 (PCL-5), depression, anxiety and adjustment disorders, supporting its validity.

## Method

### Participants and procedure

Data were collected using iPanel, a probability-based online panel consisting of more than 100 000 adults aged 18–85 years. At T1 (11 February to 17 April 2024; the duration from 7 October ranged from 4 to 6 months), 4097 participants responded of 13 694 invited (response rate: 30%). At T2 (15 August to 3 November 2024; the duration from 7 October ranged from 10 to 13 months since the event and from 6 to 9 months after T1), data collection was terminated after 2005 of the original respondents had completed the follow-up (49.0% of T1 participants). Participants were recruited through stratified sampling methods, adhering to the European Society for Opinion and Market Research international standards. A quota sampling approach, aligned with Israeli census data on geographic region, age and sex, ensured a representative sample of the general population. Notably, the Israeli population has been subjected to sustained stress over the past 2 years, because of the ongoing conflict and the pervasive threat of missile attacks throughout the country. The final data-set was weighted for age and sex, and written informed consent was obtained from all participants. The study targeted the general Israeli population aged 18–65 years who were fluent in Hebrew and had varying levels of exposure to traumatic events.

The authors assert that all procedures contributing to this work comply with the ethical standards of Israel and institutional committees on human experimentation and with the Helsinki Declaration of 1975, as revised in 2013. All procedures involving human participants were approved by the Ariel University institutional review board/committee (approval number AU-SOC-MBE-20231121).

### Measures

For all the measures, we used the official Hebrew translations of the scales; all of them went through the process of translation and back-translation by native Hebrew and native English speakers who are mental health professionals.

Subjective perception of trauma was measured using the STO scale to assess individuals’ subjective experiences of psychological trauma (STO; 1). This five-item scale measures the personal perception of psychological trauma (e.g. ‘Looking on your condition, do you feel that you suffer from psychological trauma?’), with respondents providing their answers on a five-point Likert scale ranging from ‘1’ (not at all) to ‘5’ (very much). The total STO score reflects the intensity of the subjective impact of psychological trauma, with scores ranging from 5 to 25. Cronbach’s alpha coefficients of the STO items across both waves in the current sample were 0.92.

Anxiety was measured using the International Anxiety Questionnaire (IAQ).^12^ This is a self-report instrument aligned with the ICD-11 criteria for generalised anxiety disorder (ICD-11 diagnostic code 6B00). The IAQ consists of eight items assessing symptoms of anxiety, such as excessive worry, restlessness or difficulty concentrating, experienced over the past several months (e.g. ‘Felt nervous or anxious?’). Each item is rated on a five-point Likert scale ranging from 0 (‘never’) to 4 (‘every day’), resulting in a total score between 0 and 32. Higher scores reflect greater severity of anxiety symptoms. The IAQ is based on the ICD-11 diagnostic framework, emphasising key symptoms rather than relying on cut-off scores for diagnosis. It has been validated in various clinical and research contexts and is commonly used alongside other assessments to measure the severity and impact of anxiety symptoms. Cronbach’s alpha coefficients were 0.94 and 0.95 for the wave 1 and wave 2 samples, respectively.

Depression was measured by the International Depression Questionnaire (IDQ).^12^ This is a self-report measure designed to assess symptoms of depression in accordance with ICD-11 criteria for a single episode of depressive disorder (ICD-11 diagnostic code 6A70). The IDQ comprises nine items evaluating an individual’s feelings, thought, and behaviours over the previous 2 weeks (e.g. ‘Felt down or depressed for most of the day?’). Responses are scored on a five-point Likert scale ranging from 0 (‘never’) to 4 (‘every day’), yielding a total score between 0 and 36. Higher scores indicate more severe depressive symptoms. Instead of using a cut-off score, the IDQ applies the ICD-11 diagnostic algorithm to identify cases. The tool has been extensively used in general populations and research settings, with ongoing validation efforts supporting its effectiveness across diverse populations.^12^ Cronbach’s alpha coefficients for the IDQ items across both waves in the current sample were 0.93.

Probable PTSD or CPTSD on the basis of the ICD-11 proposed criteria was measured using the ITQ.^13^ The ITQ includes six PTSD items and six items related to disturbances in self-organisation (DSO). The PTSD symptom clusters of re-experiencing, avoidance and sense of threat are measured using two items each. The DSO symptom clusters of affective dysregulation, negative self-concept and disturbances in relationships are measured by two items each. Each condition is evaluated by three items addressing functional impairment associated with the symptoms in question. Responses a measured on a scale from 0 (‘not at all’) to 4 (‘extremely’), with scores ranging from 0 to 24. Elevated risk for PTSD was computed using the algorithm provided in the original formulation of the ITQ.^13^ Cronbach’s alpha coefficients for the items across both waves in the current sample were 0.94.

Probable PTSD was assessed using the PCL-5,^14^ a 20-item self-report measure that evaluates PTSD symptoms on the basis of proposed DSM-5 criteria. Each item is rated on a five-point Likert scale, ranging from 0 (‘not at all’) to 4 (‘extremely’). The total severity score is calculated by summing the items, with possible scores ranging from 0 to 80. There are two main ways to use the PCL-5 for assessing PTSD. A provisional PTSD diagnosis can be made by considering each item rated as 2 (‘moderately’) or higher as a symptom endorsement; this can be followed by application of the DSM-5 diagnostic criteria, which require at least one symptom from the B cluster (items 1–5), one symptom from the C cluster (items 6–7), two symptoms from the D cluster (items 8–14) and two symptoms from the E cluster (items 15–20). Cronbach’s alpha coefficients for the items across both waves in the current sample were 0.95.

Adjustment disorder was measured using the International Adjustment Disorder Questionnaire (IADQ).^15,16^ This tool is structured in three sections: a psychosocial stressor checklist, a symptom scale and a functionality scale. These are all aligned with the ICD-11 core diagnostic criteria for adjustment disorder, which include stressor exposure, preoccupation with the stressor, difficulty adapting, symptom onset timing and functional impairment. The IADQ includes 19 items, with items 1 to 10 formatted as yes/no responses assessing specific stressors (e.g. financial issues such as difficulty paying bills and debts). Items 11 to 19 are rated on a five-point Likert scale ranging from 0 (‘never’) to 5 (‘very much’), with six items measuring symptom frequency over the past month and the final three items evaluating functional impairment. Cronbach’s alpha coefficients were 0.92 for the wave 1 sample and 0.93 for the wave 2 sample.

Negative worldviews were measured using a modified version of the Worldviews Scale.^17,18^ In this version, three original items were retained, and the fourth was replaced with ‘The world is a dangerous place to live in’, which is better suited to secular contexts. The measure shows good psychometric properties across populations/events.^18^ Responses are rated on a five-point Likert scale, ranging from 1 (‘never’) to 5 (‘very much’), with higher scores indicating greater negative war-related beliefs. Cronbach’s alpha coefficients were 0.85 for the wave 1 sample and 0.86 for the wave 2 sample.

The Worldviews Scale and the STO have certain similarities with respect to capture of subjective self-perception; however, there is no overlap between them, as their items assess different aspects of subjective perception.

### Data analysis

Descriptive statistics and exploratory factor analysis (EFA) were used to examine the factor structure of the STO. EFA estimated one- to three-factor solutions using robust maximum likelihood estimation with oblique geomin rotation. Standard criteria were employed to determine the optimal number of factors, with model selection guided by the Akaike information criterion and Bayesian information criterion (BIC); lower values indicated better model fit. Overall model fit and parameter estimates were evaluated, with an emphasis on model parsimony and interpretability.

Overall model fit was determined via standard guidelines,^19^ according to which good model fit is indicated by a non-significant chi-squared (*χ* ^2^) result, comparative fit index (CFI) and Tucker–Lewis index (TLI) values close to 1, and root-mean-square error of approximation (RMSEA) and standardised root-mean-square residual (SRMR) values close to 0. RMSEA and SRMR values are categorised as follows: ≤0.06 (excellent), ≤0.08 (acceptable) or ≤0.10 (marginally acceptable).^20^ The interpretability of the models was further assessed by examination of model parameters, including factor loadings and factor correlations, to ensure theoretical and practical relevance.

Validity was assessed on the basis of bivariate correlations between the STO and trauma-related measures (depression, anxiety, adjustment disorder, ITQ and PCL-5). The WAS has some similarities to the STO, particularly for the purpose of convergent validity testing. Receiver operating characteristic (ROC) analyses were used to compare the five-item and four-item version of the STO with binary PTSD/CPTSD diagnostic status (0, no diagnosis; 1, meets criteria). Youden’s index was used to determine optimal cut-offs, with values <0.70 indicating poor accuracy, and those of 0.70–0.80, 0.80–0.90, and 0.90–1.00 indicating fair, good and excellent discrimination, respectively.^21^ Sensitivity, specificity, positive and negative predictive values, and accuracy were also calculated. Analyses were conducted using R software 2025 for Windows (R Core Team, Vienna, Austria; https://www.Rproject.org/) and IBM SPSS Statistics version 29.0.2. for Windows (IBM Corp, Armonk, USA; www.ibm.com.

## Results

### STO item scores

The mean item scores for the STO in the sample for both waves are reported in Table 1. The STO item with the highest mean score in both waves was item 2, whereas the item with the lowest mean score for both waves was item 3.

**Table 1:** Descriptive statistics for the Subjective Traumatic Outlook (STO) questionnaire

STO items^a^	Mean	s.d.
Wave 1 (*N* = 4097)		
1. Looking on your condition, do you feel that you suffer from psychological trauma?	1.80	1.09
2. Looking back, do you see a fracture line between your life before the event and after the event?	2.19	1.29
3. Do you feel that the event controls your life?	1.77	1.06
4. Parallel to your daily functioning, do you feel that there is a debilitated inner world that will never recuperate from the trauma?	1.93	1.19
5. Do you feel that since the traumatic event no one can understand what you are going through?	1.82	1.19
Wave 2 (*N* = 2005)		
1. Looking on your condition, do you feel that you suffer from psychological trauma?	1.90	1.16
2. Looking back, do you see a fracture line between your life before the event and after the event?	2.32	1.33
3. Do you feel that the event controls your life?	1.82	1.10
4. Parallel to your daily functioning, do you feel that there is a debilitated inner world that will never recuperate from the trauma?	2.02	1.23
5. Do you feel that since the traumatic event no one can understand what you are going through?	1.85	1.19

a. For both waves, all item scores ranged from 0 to 5; total STO scores ranged from 0 to 25.

### EFA results for the STO: wave 1

The model fit indices indicated that a two-factor solution should be extracted (Table 2). The two-factor model provided superior fit, with *χ* ^2^ statistics significantly lower compared with those of the one-factor model (Δ*χ* ^2^(Δd.f. = 1) = 14.58; *P* < 0.001). In addition, the RMSEA and SRMR values were closer to zero, the two-factor model had a much lower residual (0.005 *v*. 0.020), and the TLI was closer to 1 for both models but that of the two-factor model was slightly better (0.991 *v*. 0.977). Finally, the BIC was lower for the two-factor model, with a difference greater than 100 points (6.26 *v*. 129.75), strongly favouring the two-factor model.

**Table 2:** Exploratory factor analysis (EFA) model fit results for the Subjective Traumatic Outlook (STO) questionnaire

EFA^a^	*χ* ^2b^	d.f.	*P*-value	TLI	RMSEA (90% CI)	SRMR	BIC
Wave 1 STO							
Model 1: unidimensional model	171.34	5	<0.001	0.977	0.090	0.020	129.75
Model 2: two factors	14.58	1	<0.001	0.991	0.058	0.005	6.26
Wave 2 STO							
Model 1: unidimensional model	168.03	5	<0.001	0.978	0.089	0.020	126.44
Model 2: two factors	4.80	1	0.028	0.997	0.030	0.003	−3.52

TLI, Tucker–Lewis index; RMSEA, root-mean-square error of approximation; SRMR, standardised root-mean-square residual; BIC, Bayesian information criterion.

a. First-order factors are latent factors measured by observed variables.

b. Chi-squared goodness-of-fit statistic.

The standardised loadings generated for the two-factor solution are presented in Table 3. Factor one was labelled as ‘Intrapersonal perceptual impact’ and included items 1**–**4 The second factor was labelled as ‘social-intersubjective’ and included only item 5. All item loadings were high, positive and statistically significant (*P* < 0.01). The correlations between factors were high and had strong loadings (>0.7) on their primary factors. The correlations between factors were high at both time points (0.77).

**Table 3:** Item mapping for exploratory factor analysis (EFA)-suggested alternative factor models for the Subjective Traumatic Outlook (STO) questionnaire

STO items: EFA	Factor 1 Intrapersonal perceptual impact	Factor 2 Interpersonal, social experience
Wave 1		
1. Looking on your condition, do you feel that you suffer from psychological trauma?	**0.795**	–
2. Looking back, do you see a fracture line between your life before the event and after the event?	**0.917**	–
3. Do you feel that the event controls your life?	**0.838**	–
4. Parallel to your daily functioning, do you feel that there is a debilitated inner world that will never recuperate from the trauma?	**0.743**	–
5. Do you feel that since the traumatic event no one can understand what you are going through?	–	**0.981**
Wave 2		
1. Looking on your condition, do you feel that you suffer from psychological trauma?	**0.753**	–
2. Looking back, do you see a fracture line between your life before the event and after the event?	**0.958**	–
3. Do you feel that the event controls your life?	**0.723**	–
4. Parallel to your daily functioning, do you feel that there is a debilitated inner world that will never recuperate from the trauma?	**0.600**	0.331
5. Do you feel that since the traumatic event no one can understand what you are going through?	–	**0.797**

Bold text denotes the study variables of primary interest.

### EFA results for the STO: wave 2

As for wave 1, the two-factor solution provided a better model fit than the one-factor solution (Δ*χ* ^2^(1) = 4.80, *P* < 0.001; Table 2). The RMSEA and SRMR values were again lower for the two-factor model (0.030 *v*. 0.089), and the TLI was slightly improved (0.997 *v*. 0.978). The BIC values showed a strong preference for the two-factor model (−3.52 *v*. 126.44), indicating continued model preference.

The factor structure remained consistent across waves. As shown in Table 3, factor 1 encompassed the same four items noted above, whereas factor 2 continued to include the single item regarding perceived incomprehensibility by others. All loadings were statistically significant (*P* < 0.01), with strong primary factor loadings (>0.70). The correlation between factors was slightly higher in wave 2 (*r* = 0.82), indicating stable inter-factor relationships over time. Notably, item 4 (‘a debilitated inner world that will never recuperate’) showed minor cross-loading in the second wave. The effect sizes for the STO items from T1 to T2 are presented in Table 4.

**Table 4:** Waves 1 and 2: *t*-test analysis model fit results for the Subjective Traumatic Outlook (STO) questionnaire

STO items	T1 Mean (s.d.)	T2 Mean (s.d.)	*t* (d.f.)	*P*-value	Cohen’s *d*
STO 1	1.84 (1.11)	1.90 (1.16)	−3.03 (2003)	0.002	−0.070
STO 2	2.23 (1.30)	2.32 (1.33)	−3.65 (2003)	<0.001	−0.082
STO 3	1.78 (1.07)	1.82 (1.10)	−2.19 (2003)	0.029	−0.050
STO 4	1.94 (1.19)	2.02 (1.23)	−3.32 (2003)	<0.001	−0.074
STO 5	1.84 (1.20)	1.85 (1.91)	−3.20 (2003)	0.750	−0.007
STO total score	9.63 (5.14)	9.91 (5.26)	−3.36 (2003)	<0.001	−0.075

### Bivariate correlation matrix

The STO demonstrated consistently moderate-to-strong correlations with all measures across both time points. Specifically, STO scores showed strong associations with DSM-5 PTSD symptoms (PCL-5; *r* = 0.73–0.77), depression (*r* = 0.66–0.69) and anxiety (*r* = 0.66–0.69). Moderate correlations were observed with ICD-11 PTSD symptoms (ITQ; *r* = 0.57–0.62), disturbances in self-organisation (*r* = 0.53–0.62), adjustment disorder symptoms (IADQ; *r* = 0.62–0.66) and the Worldviews questionnaire (*r* = 0.57–0.60). This pattern of correlations suggests that although the STO has conceptual overlap with existing measures, it captures unique dimensions of trauma-related experience. Complete correlation matrices are presented in Table 5.

**Table 5:** Intercorrelations (*r*) among study variables^a^

Variable	1	2	3	4	5	6	7	8	9	10	11	12	13	14	15	16	17	18
1. T1 STO (five items) score	–																	
2. T1 STO (four items) score	0.90^**^	–																
3. T1 PTSD symptoms^b^	0.57^**^	0.57^**^	–															
4. T1 DSO symptoms only^c^	0.62^**^	0.60^**^	0.52^**^	–														
5. T1 PCL-5 total	0.74^**^	0.73^**^	0.78^**^	0.73^**^	–													
6. T1 IADQ	0.62^**^	0.62^**^	0.71^**^	0.56^**^	0.75^**^	–												
7. T1 depression^d^	0.67^**^	0.66^**^	0.59^**^	0.71^**^	0.85^**^	0.66^**^	–											
8. T1 anxiety^e^	0.67^**^	0.66^**^	0.64^**^	0.67^**^	0.88^**^	0.69^**^	0.86^**^	–										
9. T1 worldviews	0.57^**^	0.57^**^	0.59^**^	0.60^**^	0.69^**^	0.70^**^	0.64^**^	0.65^**^	–									
10. T2 STO (five items) score	0.74^**^	0.74^**^	0.59^**^	0.56^**^	0.66^**^	0.54^**^	0.62^**^	0.61^**^	0.52^**^	**–**								
11. T2 STO (four items) score	0.73^**^	0.73^**^	0.54^**^	0.51^**^	0.65^**^	0.53^**^	0.61^**^	0.61^**^	0.53^**^	0.99^**^	–							
12. T2 PTSD symptoms^b^	0.53^**^	0.54^**^	0.69^**^	0.44^**^	0.68^**^	0.63^**^	0.57^**^	0.61^**^	0.53^**^	0.61^**^	0.62^**^	–						
13. T2 DSO symptoms only^c^	0.54^**^	0.52^**^	0.41^**^	0.74^**^	0.62^**^	0.44^**^	0.61^**^	0.57^**^	0.49^**^	0.53^**^	0.53^**^	0.53^**^	–					
14. T2 PCL total	0.65^**^	0.64^**^	0.62^**^	0.64^**^	0.80^**^	0.63^**^	0.74^**^	0.75^**^	0.62^**^	0.77^**^	0.76^**^	0.80^**^	0.75^**^	–				
15. T2 IADQ	0.54^**^	0.54^**^	0.54^**^	0.47^**^	0.69^**^	0.69^**^	0.58^**^	0.62^**^	0.70^**^	0.65^**^	0.66^**^	0.73^**^	0.58^**^	0.76^**^	–			
16. T2 depression^d^	0.59^**^	0.59^**^	0.50^**^	0.64^**^	0.73^**^	0.57^**^	0.71^**^	0.71^**^	0.58^**^	0.69^**^	0.68^**^	0.61^**^	0.75^**^	0.87^**^	0.68^**^	–		
17. T2 anxiety^e^	0.59^**^	0.58^**^	0.55^**^	0.59^**^	0.74^**^	0.60^**^	0.71^**^	0.76^**^	0.58^**^	0.69^**^	0.68^**^	0.66^**^	0.68^**^	0.88^**^	0.72^**^	0.88^**^	–	
18. T2 worldviews	0.51^**^	0.52^**^	0.50^**^	0.53^**^	0.63^**^	0.60^**^	0.59^**^	0.59^**^	0.72^**^	0.59^**^	0.60^**^	0.61^**^	0.60^**^	0.71^**^	0.66^**^	0.66^**^	0.66^**^	–

STO, Subjective Traumatic Outlook questionnaire; PTSD, post-traumatic stress disorder; DSO, disturbances in self-organisation; PCL-5, PTSD Checklist for DSM-5; IADQ, International Adjustment Disorder Questionnaire.

a. *N* = 1303–4097. Variables with smaller sample sizes (*N* = 1303–2086) include subset analyses (variables 10–18).

b. International Trauma Questionnaire (ITQ) items P1–P6.

c. ITQ items C1–C6.

d. International Depression Questionnaire symptoms.

e. International Anxiety Questionnaire symptoms.

** *P* < 0.01.

Bold text denotes the study variables of primary interest.

### ROC analysis for the five-item STO and PTSD

ROC analysis was conducted to establish a clinically useful cut-off point for the five-item STO scale using binary PTSD diagnostic status (ICD-11 criteria: 0, not meeting criteria; 1, meeting criteria). Youden’s index reached its maximum at a cut-off value of 9.5, indicating a significantly higher likelihood of probable PTSD diagnosis in individuals scoring ≥9.5.

**Fig. 1:**
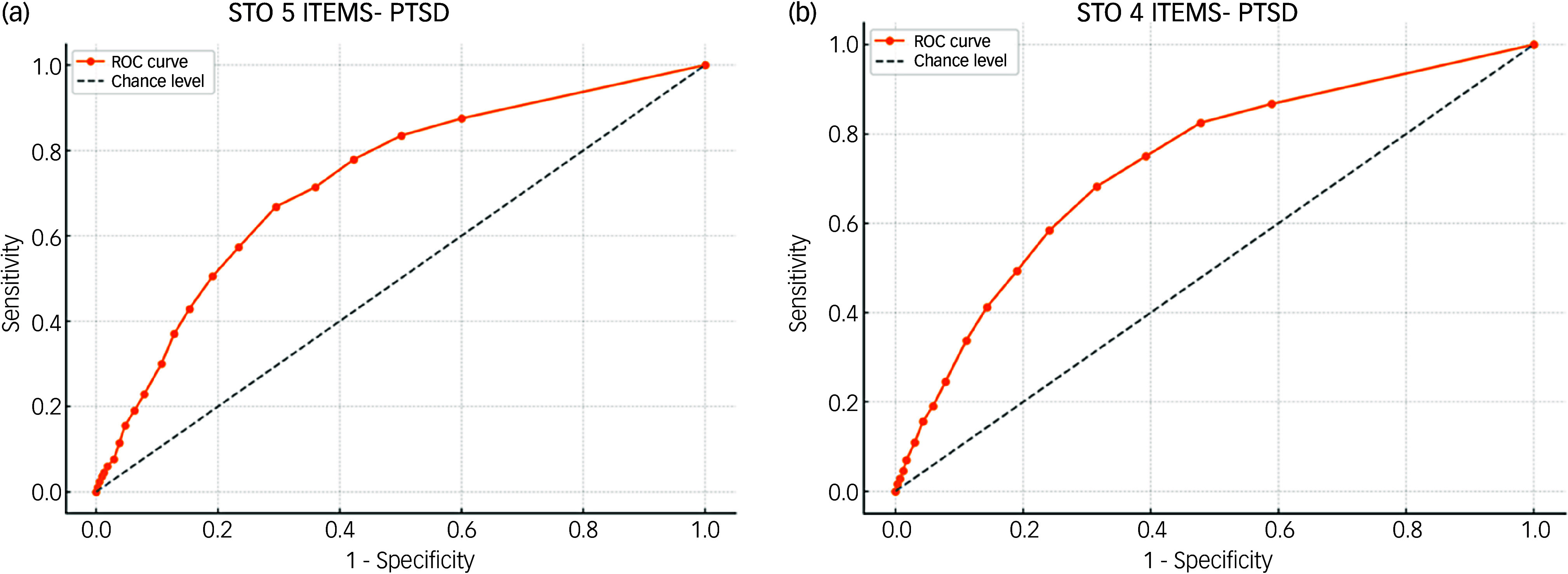
Receiver operating characteristic (ROC) curves for the Subjective Traumatic Outlook (STO) questionnaire (five versus four items) and post-traumatic stress disorder (PTSD).

At this cut-off, the STO demonstrated a sensitivity of 66.80% (95% CI [62.49%, 70.90%]) and specificity of 70.49% (95% CI [68.87%, 72.07%]). The positive predictive value was 26.29% (95% CI [24.74%, 27.09%]), and the negative predictive value was 93.09% (95% CI [92.24%, 93.86%]). Positive and negative likelihood ratios were 2.26 (95% CI [2.09, 2.46]) and 0.47 (95% CI [0.42, 0.53]), respectively. The overall accuracy was 69.99% (95% CI [68.48%, 71.46%]), with an estimated prevalence of 13.61% (95% CI [12.52%, 14.76%]). Complete ROC analysis results are presented in Fig. 1(a).

### ROC analysis for the four-item STO and PTSD

ROC analysis was conducted to establish a clinically useful cut-off point for the four-item STO scale using binary PTSD diagnostic status (ICD-11 criteria: 0, not meeting criteria; 1, meeting criteria). Youden’s index reached its maximum at a cut-off value of 7.5, indicating a significantly higher likelihood of probable PTSD diagnosis in individuals scoring ≥7.5.

At this cut-off, the STO demonstrated a sensitivity of 68.19% (95% CI [63.92%, 72.24%]) and specificity of 68.48% (95% CI [66.84%, 70.09%]). The positive predictive value was 25.43% (95% CI [23.96%, 26.95%]), and the negative predictive value was 93.18% (95% CI [92.31%, 93.96%]). Positive and negative likelihood ratios were 2.16 (95% CI [2.00, 2.34]) and 0.46 (95% CI [0.41, 0.53]), respectively. The overall accuracy was 68.44% (95% CI [66.92%, 69.94%]), with an estimated prevalence of 13.61% (95% CI [12.52%, 14.76%]). Complete ROC analysis results are presented in Fig. 1(b).

### ROC analysis for the five-item STO and CPTSD

ROC analysis was conducted to establish a clinically useful cut-off point for the five-item STO scale using binary CPTSD diagnostic status (ICD-11 criteria: 0, not meeting criteria; 1, meeting criteria). Youden’s index reached its maximum at a cut-off value of 11.5, indicating a significantly higher likelihood of probable CPTSD diagnosis in individuals scoring ≥11.5.

**Fig. 2:**
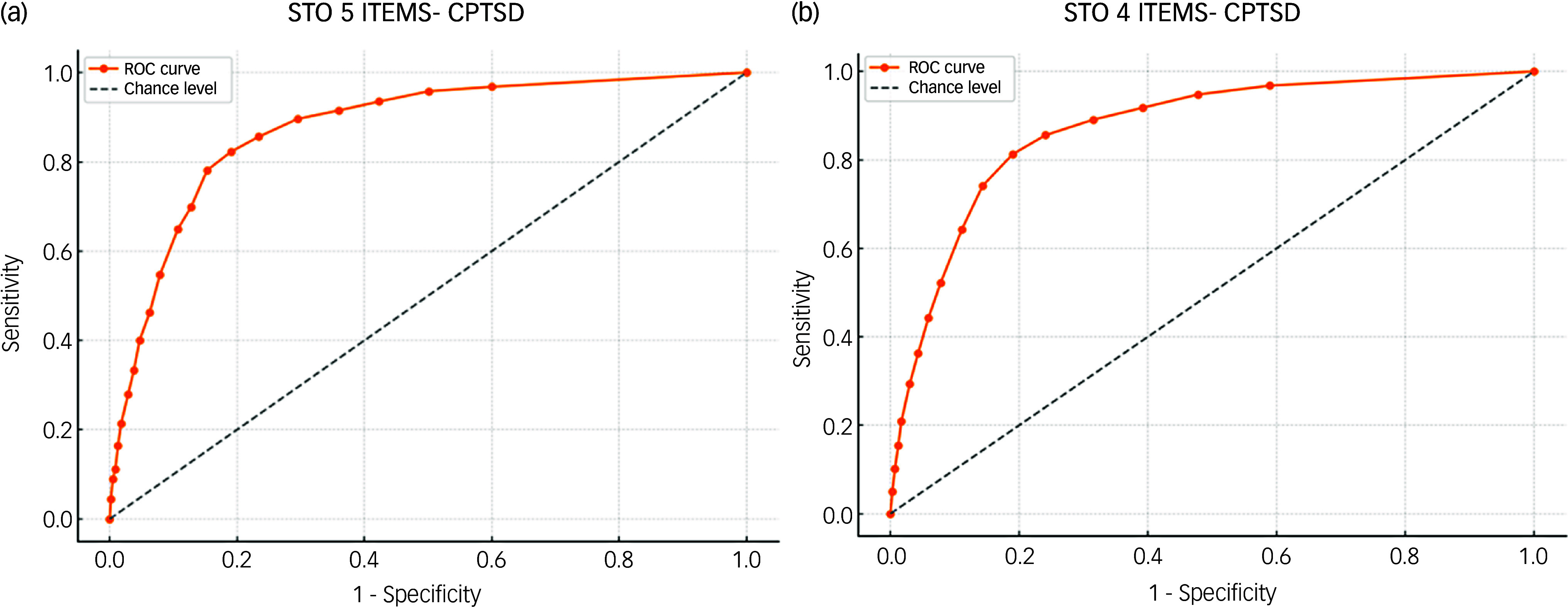
Receiver operating characteristic (ROC) curves for the Subjective Traumatic Outlook (STO) questionnaire (five versus four items) and complex post-traumatic stress disorder (CPTSD).

At this cut-off, the STO demonstrated a sensitivity of 82.34% (95% CI [78.25%, 85.94%]) and specificity of 80.86% (95% CI [79.45%, 82.21%]). The positive predictive value was 35.14% (95% CI [33.24%, 37.09%]), and the negative predictive value was 97.32% (95% CI [96.71%, 97.82%]). Positive and negative likelihood ratios were 4.30 (95% CI [3.95, 4.68]) and 0.22 (95% CI [0.18, 0.27]), respectively. The overall accuracy was 81.02% (95% CI [79.70%, 82.29%]), with an estimated prevalence of 11.19% (95% CI [10.17%, 12.26%]). Complete ROC analysis results are presented in Fig. 2(a).

### ROC analysis for the four-item STO and CPTSD

ROC analysis was conducted to establish a clinically useful cut-off point for the four-item STO scale using binary CPTSD diagnostic status (ICD-11 criteria: 0, not meeting criteria; 1, meeting criteria). Youden’s index reached its maximum at a cut-off value of 9.5, indicating a significantly higher likelihood of probable CPTSD diagnosis in individuals scoring ≥9.5.

At this cut-off, the STO demonstrated a sensitivity of 81.34% (95% CI [77.18%, 85.03%]) and specificity of 81.05% (95% CI [79.64%, 82.39%]). The positive predictive value was 35.09% (95% CI [33.16%, 37.06%]), and the negative predictive value was 97.18% (95% CI [96.56%, 97.69%]). Positive and negative likelihood ratios were 4.29 (95% CI [3.94, 4.68]) and 0.23 (95% CI [0.19, 0.28]), respectively. The overall accuracy was 81.08% (95% CI [79.76%, 82.35%]), with an estimated prevalence of 11.19% (95% CI [10.17%, 12.26%]). Complete ROC analysis results are presented in Fig. 2(b).

## Discussion

This study aimed to validate the STO scale within the context of the Israeli population exposed to the 7 October attacks. The STO demonstrated strong psychometric properties, with robust internal consistency and construct validity across two waves of data collection. EFA analyses supported a two-factor structure across both measurements: factor 1 (four-item: intrapersonal perceptual impact) and factor 2 (one item: social-intersubjective).

The near-identical factor loadings and fit indices across measurements provide preliminary evidence of the stability of the STO over two time points after the attack. These results were consistent with previous findings^2,3^ and offer a systematic over-time evaluation of the underlying factor structure of the STO. The minor cross-loading of item 4 (‘Parallel to your daily functioning, do you feel that there is a debilitated inner world that will never recuperate from the trauma’) in wave 2 may reflect a stage at which internal disruption begins to affect daily functioning. This aligns with Janoff-Bulman’s^22^ notion of shattered assumptions, wherein traumatic experiences precipitate a revaluation of core beliefs about safety, predictability and self-identity.

Notably, the consistency of item rankings across both waves reinforced the stability of respondents’ perceptions: item 2 (‘fracture line’) consistently yielded the highest mean, whereas item 3 (‘event controls your life’) remained the lowest. The consistency of item 2 in terms of having the highest mean suggests that perceiving life as divided before versus after the trauma is a central feature of subjective trauma outlook across time. This item may represent an ego-dystonic experience,^4^ signalling distress that conflicts with one’s pre-trauma self-concept and contributes to cognitive dissonance as individuals struggle to reconcile their current psychological state with their prior sense of identity.

Moreover, the emergence of a stable two-factor solution across both waves indicated that the STO captures two related but distinct dimensions: first, the core experiential and/or perceptual impact of trauma (factor 1); and, second, the social-intersubjective sense of being misunderstood (factor 2). Although these factors are empirically distinguishable, their high inter-factor correlations may suggest they are facets of a broader latent construct related to subjective trauma interpretation. Factor 2, anchored by the item reflecting a profound sense of social isolation (‘no one can understand what you are going through’), may capture an element of post-traumatic loneliness. This dimension is increasingly recognised as relevant in the context of PTSD.^23–25^ However, this item diverges conceptually from the remaining STO items, which primarily tap into subjective internal experiences of trauma, rather than interpersonal disconnection. This potential divergence highlights the need for further evaluation of the structure of the scale and the conceptual aspects of individual items.

Bivariate correlations indicated that all associations were statistically significant yet not excessively overlapping; this suggests that the STO measures a distinct construct, further supporting its differentiation from instruments such as the WAS. The fifth STO item showed limited added value, as correlation patterns between the five-item and four-item versions were almost identical across psychological measures, indicating minimal utility of the additional item. Moreover, ROC results indicated that removing the fifth item did not affect diagnostic accuracy.

Similarly, for probable CPTSD, the four-item version performed equivalently to the five-item scale, with sensitivity, specificity and overall accuracy exceeding 81% in both models. These findings indicate that the fifth item does not meaningfully enhance the predictive validity of the STO for either PTSD or CPTSD diagnosis. The results thus suggest limited diagnostic value of the fifth item.

The STO offers an efficient, scalable screening tool well-suited to rapid psychological assessment in high-demand settings, such as wartime. Its brevity and focus support timely triage and targeted interventions, helping to identify at-risk individuals for appropriate care. The prominence of the ‘fracture line’ perception underscores the potential utility of narrative-based therapies that explicitly address temporal discontinuity in the life stories of trauma survivors.

This study provides one of the first examinations of subjective trauma perceptions through cross-sectional assessment of the psychometric properties of the STO in a wartime context. However, several limitations should be considered. First, reliance on self-reported data introduces potential response biases and may not fully capture the complexity of trauma experiences. Second, data collection occurred online during the immediate aftermath of the 7 October attack, which may limit generalisability to other trauma types (e.g. natural disasters, chronic stressors) or different temporal phases of trauma response. Third, the absence of clinical diagnostic interviews constrains validation of symptom assessments against gold-standard clinical criteria

Finally, the identification of a single-item factor is problematic, as factors comprising only one item lack psychometric validity and fail to represent true latent constructs. Single-item factors cannot demonstrate internal consistency and may reflect measurement error rather than meaningful psychological dimensions. Future research should evaluate whether removal of this item to create a four-item STO or expansion of the social-intersubjective dimension with additional items would result in a more robust factor structure with enhanced psychometric properties and diagnostic utility. Although the tool demonstrates strong psychometric properties, including a stable two-factor solution across both waves within a high-stress context, and our findings support its conceptual depth and its clinical relevance, future research should also further evaluate its psychometric properties in more diverse populations and settings to enhance its utility as a brief, targeted for clinical applications. We also recommend the use of the STO in conflict settings as a predictor of subsequent ICD-11 stress-related disorders.

## Data Availability

Any request concerning the data should be made directly to M.B.-E. (menbe@ariel.ac.il) or Y.L. (yafitl@ariel.ac.il). OSF link: https://osf.io/yd5tu/?view_only=d7ef4b402afa4ecfbe829a6ba7bc23b1.
